# Design and Synthesis of a Novel Cationic Peptide with Potent and Broad-Spectrum Antimicrobial Activity

**DOI:** 10.1155/2015/578764

**Published:** 2015-11-25

**Authors:** Wen-Ping Liu, Ya-Hui Chen, Xin Ming, Yi Kong

**Affiliations:** ^1^Children's Hospital of Zhengzhou, Zhengzhou 450053, China; ^2^School of Life Science and Technology, China Pharmaceutical University, 24 Tong Jia Street, Nanjing 210009, China; ^3^Division of Molecular Pharmaceutics, UNC Eshelman School of Pharmacy, The University of North Carolina at Chapel Hill, Chapel Hill, NC 27599, USA

## Abstract

Antibacterial and antifungal peptides have increasingly been used to combat the antibiotic-resistant microbes in recent years. KW-13, a novel cationic *α*-helical antibacterial peptide consisting of 13 amino acid residues, was designed and chemically synthesized. The peptide has a net charge of +6 with a total hydrophobic ratio of 38%. The antibacterial experiments revealed that KW-13 strongly inhibited the growth of human pathogenic bacteria with minimal inhibitory concentrations of 4 and 16 *μ*g/mL for* Staphylococcus epidermidis* and* Staphylococcus aureus,* respectively, while the hemolytic assay showed that this peptide did not destroy human red blood cells* in vitro*. Scanning electron microscopy imaging of* Escherichia coli* confirmed that KW-13 can damage the membrane of bacterial cells. Thus, this peptide could be a potential candidate for the treatment of infectious diseases.

## 1. Introduction

Drug resistance to microorganisms, including bacteria such as* staphylococci, enterococci* and* Escherichia coli,* fungi, viruses and parasites, has become a major challenge for human health worldwide. Antimicrobial peptides (AMPs) are good candidates as new antibiotics since they are natural defenses of most living organisms against invading pathogens [1–3]. Some of them have also been found showing antitumor and spermicidal activities [4, 5]. These AMPs are relatively small (<10 kDa in MW), cationic, and amphipathic peptides with variable length, sequence, and structure. The first AMP cecropin was discovered from* Hyalophora cecropia* in 1981 [6]. In the following years, peptides with antibacterial activity have been found in the whole natural kingdom, from bacteria, insects, and amphibians to mammals [7–9]. To exert antimicrobial action, they use a common “carpet” mechanism, in which they accumulate on the bacterial membrane up to a threshold concentration, and then cause membrane permeabilization/disintegration [10].

The AMPs are classified into five groups [11]: (1) peptides that form *α*-helical structure, (2) peptides that are rich in cysteine residues, (3) peptides that form *β*-sheet structure, (4) peptides that are rich in regular amino acids, namely, histidine, arginine, and proline, and (5) peptides that are composed of rare and modified amino acids. These general features can be used as guidance for design and synthesis of potent AMPs.

The natural AMPs are far from perfect and some of them cause toxicity to the eukaryotes. For example, the strong antibacterial activity is often accompanied with hemolysis to the eukaryotes. So the main purpose of this study is to improve their antibacterial activity and reduce toxicity to eukaryotes. Rational design and thereafter chemical synthesis are important approaches in the search for substitutes of conventional antibiotics because it can overcome the limitations in large-scale production and application of natural peptides, such as the low specific activity and the small amounts recovered from natural sources. There are many databases and software for active peptide design. The website at http://web.expasy.org/blast/ is a useful tool for sequence analysis and secondary structure prediction. Accelrys Insight II provides powerful function for studying structure and target-based drug design. Antimicrobial Peptide Database (APD) is a specialized database containing many antimicrobial peptides and it provides query, prediction, and design of peptides [12, 13]. It has been established that the physicochemical properties of AMPs, including secondary structure, overall charge and hydrophobicity, affect their interaction with model membranes and mammalian cells [14–17]. Most of AMPs fold into an amphipathic *α*-helical conformation when interacting with the target microorganism. With the development of these structure-activity relationship and mechanism research, it is feasible to design AMPs, which are more efficient to kill microorganisms and less toxic to eukaryotes.

In this study, we designed and synthesized six peptides based on characteristics of natural AMPs that are preferably cationic, with *α*-helix, and with more than 30% of hydrophobic residues. One of them KW-13 showed potent inhibitory activity against bacteria without hemolytic action on human blood red cells. This peptide could be a potential candidate for the treatment of infectious diseases.

## 2. Materials and Methods 

### 2.1. Design of Peptide

The antibacterial peptides were designed using the APD (http://aps.unmc.edu/AP/main.php) [12, 13]. The peptide sequences were chosen based on the following rules: (1) containing positive charged amino acids, (2) containing *α*-helix, and (3) containing hydrophobic amino acids. Variation of the physicochemical features predicted according to this database is shown in Table 1.

### 2.2. The Strains and Growth Conditions

Two Gram-positive bacteria, 3 Gram-negative bacteria, and 2 fungi were selected to measure antibacterial activity of peptides. The strains were provided by Department of Microbiology in China Pharmaceutical University and were stored at 4°C until use. The species and culture condition of these bacterial strains are summarized in Table 3.

### 2.3. Peptide Synthesis and Purification

Peptides were synthesized by the Fmoc (N-[9-fluorenyl]-methoxycarbonyl) chemistry according to the literature procedure [18, 19]. Protected amino acids were coupled by* in situ* activation with N,N-diisopropylethylamine (DIEA) and N-hydroxybenzotriazole (HOBt). Deprotection was performed with 20% piperidine in N,N-dimethylformamide (DMF). The protected side chains of amino acid residues and the cleavage of the peptide from the solid support were performed by 95% trifluoroacetic acid (TFA)/2.5% triisopropylsilane (TIS)/2.5% water for 1 h at room temperature. After cleavage from resin, the peptides were purified by preparative reverse-phase HPLC (BioLogic Duoflow system) on a Kromasil C18 column (250 × 10 mm, particle size 5 *μ*m, pore size 100 Å). The elution was achieved with a linear gradient of 0.05% TFA in 5% methanol (A) and 0.05% TFA in 95% methanol (B) at a flow rate of 5 mL/min (57–64% B in 30 min). The main peak was pooled, lyophilized, and stored at −20°C. The purity of the peptide was evaluated using analytical reverse-phase HPLC (Shimadzu LC-10AT) on a Lichrospher C18 column (250 × 4.6 mm) in the same mobile phase with a linear gradient at a flow rate of 1 mL/min (49–54% B in 20 min). The synthetic peptides were confirmed by electrospray mass spectrometry.

### 2.4. Measurement of Antibacterial Activity

Antimicrobial activity was evaluated using an inhibition zone assay or determination of the minimal inhibitory concentration (MIC) value with minor modifications as described previously [20]:

1. Inhibition zone assay: agar plates were seeded with strains (about 10^6^ cells in 10 mL of 1% agar medium). Wells (3 mm in diameter) were punched out and a 5 *μ*L peptide sample dissolved in water was loaded. Controls were loaded with water. After incubation at 37°C overnight, the diameter of the inhibition zone was determined.
2. Determination of the MIC value: MICs were determined using a standard serial dilution method in appropriate medium for each bacterial strain listed in Table 3. In brief, 1 mL of different peptides dilution solutions was poured into a series of sterile plates, and then 9 mL appropriate medium that was preincubated at 37°C was added into a plate and was mixed gently to reach various concentrations (512, 256, 128, 64, 32, 16, 8, 4, 2, 1, and 0.5 *μ*g/mL). Bacteria were added to the 96-well plates. The amount of bacteria for each spot is approximately 10^4^ to 10^5^ CFU/mL. After incubating the inoculated plate at 37°C for 16 to 18 hours, MICs were determined as the lowest peptide concentration that inhibited bacterial growth. All MICs were determined in two independent experiments performed in duplicate.

### 2.5. Measurement of Hemolytic Activity (MHC)

Hemolytic activity was determined by the procedure of Yoshida et al. with slight modifications [21]. Fresh human blood (1 mL) was centrifuged at 1000 ×g for 5 min and the precipitates were collected and then washed 3 times with phosphate buffered saline (PBS) (pH 7.4). Precipitates were resuspended in 4-fold volumes of PBS. Then, 995 *μ*L of PBS solution containing serial diluted peptides was added to a human erythrocyte solution of 5 *μ*L. The resulting solution was incubated at 37°C for 1 h and was centrifuged at 1000 ×g for 5 min. The supernatant was diluted 5-fold with PBS and was monitored at 415 nm using a UV spectrophotometer. Zero hemolysis (blank) and 100% hemolysis were determined in PBS and 1% Triton X-100, respectively.

### 2.6. Scanning Electron Microscopy

A mid-logarithmic phase culture of* Escherichia coli* was exposed to KW-13 (256 *μ*g/mL) or water at 37°C for 2 h. Bacteria were precipitated by centrifugation at 5000 rpm for 5 min and washed 3 times in PBS (pH 7.4). The supernatants were removed and the pellets were fixed in 800 *μ*L of 2.5% glutaraldehyde in 0.1 M PBS at 4°C for 24 h. The fixed bacteria were centrifuged at 5000 rpm for 10 min, washed 2 times with 0.1 M PBS, and then step-dehydrated with 70%, 80%, 90%, and 100% ethanol. After drying and gold coating, the samples were examined by scanning electron microscopy.

### 2.7. Homology with Other Antimicrobial Peptides

**Figure 1:**
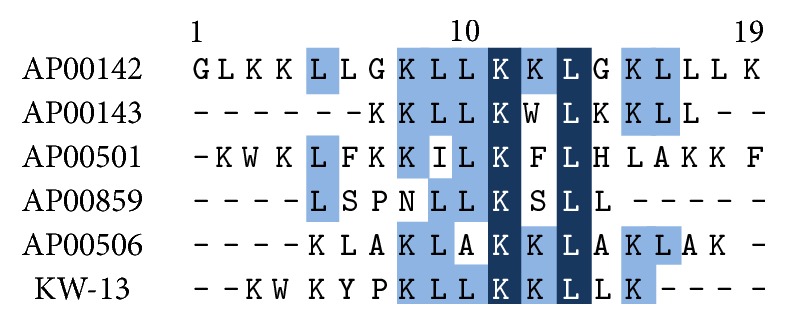
ClustalW alignment of the KW-13 with the most similar AMPs in the APD database.

A ClustalW tool was used for peptide sequences alignment [22]. The results are presented in Table 4 and Figure 1.

### 2.8. Secondary Structure Prediction

Secondary structure modeling was performed from a template model constructed by putative conserved domains associated with the primary sequence of the novel peptide. This was conducted at the NCBI server using BLAST (http://www.ncbi.nlm.nih.gov/blast/Blast.cgi). The three-dimensional structure of peptide was analyzed using the Accelrys discovery studio.

## 3. Results and Discussion

### 3.1. Peptide Design and Structure Prediction

**Figure 2:**
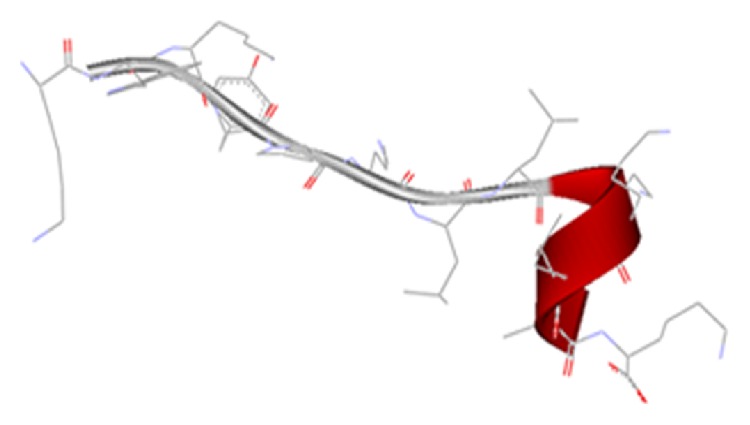
The secondary structure model for KW-13. The color codes are red for the regular *α*-helix and white for random coil.

Six peptides were designed based on characteristics of natural AMPs, including small size, cationicity, amphipathicity, and *α*-helix. Sequences of peptides and their design explanation are listed in Table 1. KW-13 consists of 13 amino acid residues, its N-terminal domain contains two cationic lysine residues, C-terminal contains a standard *α*-helix (KLLK), and these two domains are connected with a proline residue. The APD-based prediction indicated that KW-13 has 4 Leu and 1 Trp, showing a APD defined total hydrophobic ratio of 38%, and 6 Lys, showing +6 positive net charge, and that KW-13 may form alpha helices and at least 3 residues on the same hydrophobic surface. This peptide may interact with membranes and therefore becomes an antimicrobial peptide. The physicochemical properties of KW-13 are summarized in Table 2. The alignment was performed by ClustalW tool to compare the degree of homology of KW-13 with other antimicrobial peptides. The results showed that KW-13 shared similarity percentages of 61.53%, 50%, 50%, 50%, and 46.15% with known antibacterial peptides, AP00143, AP00506, AP00501, AP00142, and AP00859, respectively (Table 4 and Figure 1). The three-dimensional structure of this peptide was constructed by homology modeling using the tool of Accelrys discovery studio (Figure 2), showing that random coil and *α*-helix distributes in two well-defined zones, N-terminus and C-terminus, respectively. Analysis using the ExPASy MW/pI tool (http://web.expasy.org/compute_pi/) indicated that the PI of KW-13 is 10.40 [23]. BLAST searches (http://www.ncbi.nlm.nih.gov/blast/Blast.cgi) showed that KW-13 is a novel peptide.

### 3.2. Peptide Synthesis and Characterization

Six peptides were chemically synthesized by a solid-phase peptide synthesis method and then were purified by preparative RP-HPLC on C18 column. The purity of KW-13 was above 99% according to analytical HPLC. Its molecular weight was determined by mass spectrometer analysis and the result was consistent with the calculated molecular weight of 1686.20 Da.

### 3.3. Antimicrobial Activity

Antimicrobial activities of six peptides (KW-13, RFFR-15, RFPP-18, KWKK-13, KPV-13, and KPV-8) were investigated against several microorganisms. The MICs were determined and summarized in Table 5. RFFR-15, RFPP-18, KWKK-13, KPV-13, and KPV-8 showed weak activities against bacteria and did not show activity against fungi. Interestingly, KW-13 showed potent activity against bacteria. The MICs of KW-13 for* Staphylococcus aureus*,* Staphylococcus epidermidis*,* Escherichia coli*,* Klebsiella pneumoniae*, and* Pseudomonas aeruginosa* were comparable to ampicillin and streptomycin sulfate which have been used in clinic. The results also showed that KW-13 strongly inhibited the growth of Gram-positive bacteria (G^+^) such as* Staphylococcus aureus* (16 *μ*g/mL) and* Staphylococcus epidermidis* (4 *μ*g/mL), indicating that KW-13 was more powerful than Magainin 1, an antimicrobial peptide from Xenopus skin inhibiting* Staphylococcus aureus*,* E. coli*,* Klebsiella pneumoniae,* and* Pseudomonas aeruginosa* [24]. However, KW-13 was less effective against fungi, such as* Aspergillus niger* (256 *μ*g/mL).

### 3.4. Scanning Electron Microscopy

**Figure 3:**
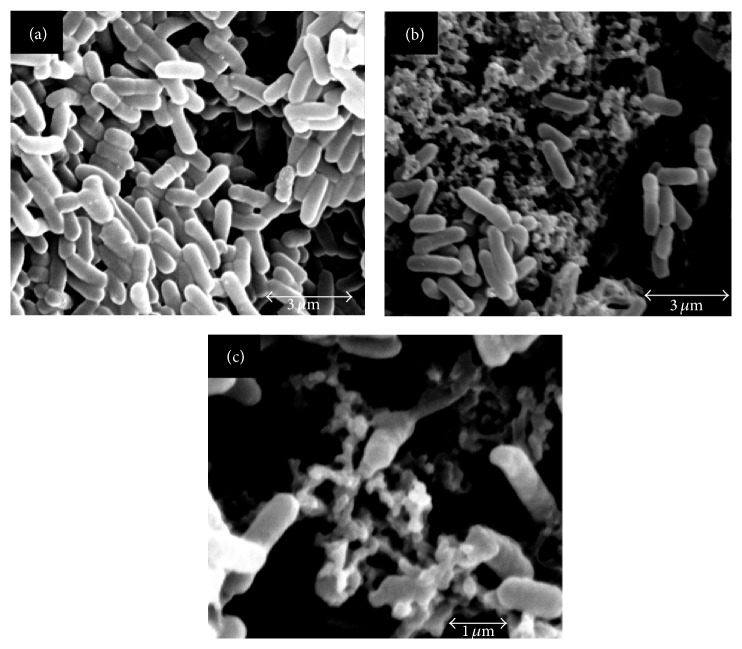
Scanning electron micrographs of* E. coli* treated with KW-13.* E. coli* in mid-logarithmic growth was incubated with antibacterial peptide KW-13 for 2.5 h. (a) Untreated* E. coli* showing a normal intact cell surface. Scale bar is 3 *μ*m. (b) Treated with 256 *μ*g/mL KW-13 for 2.5 h, the* E. coli* cell membranes were disrupted. Scale bar is 3 *μ*m. (c) The amplification of (b) in different visual field. Scale bar is 1 *μ*m.

Scanning electron microscopy was used to examine whether KW-13 can damage* E. coli* cell membranes.* E. coli* treated with KW-13 (256 *μ*g/mL) were largely destroyed and numerous cellular fragments were observed (Figures 3(b) and 3(c)), whereas the control bacteria showed normal intact surface (Figure 3(a)). It was reported that cationic AMPs with net positive charges can interact with negatively charged groups in G^−^bacteria membranes such as lipopolysaccharides, leading to the formation of “wormholes” and the collapse of the bacteria [25]. Membrane disruption can lead to leakage of ions and metabolites, depolarization, and ultimately cell death. This result indicated that KW-13 could kill* E. coli* by destroying the cell membrane.

### 3.5. Hemolytic Assay

KW-13 was challenged with hemolytic assay against human blood red cell (RBC) at 10 *μ*g/mL, 40 *μ*g/mL, 160 *μ*g/mL, and 640 *μ*g/mL. No significant hemolysis (<1%) was observed even at high concentration (640 *μ*g/mL), while a well-known hemolytic peptide, Melittin, caused the lysis of whole erythrocytes at a concentration below 40 *μ*g/mL. Our results indicated KW-13 was not toxic to human RBCs.

## 4. Conclusions

In conclusion, KW-13, a novel cationic *α*-helical antibacterial peptide, showed potent inhibitory activity against human pathogenic bacteria without destroying human red blood cells. This peptide inhibited bacterial growth by destroying the membrane of it. Further works should be performed in animal models to confirm the antibacterial function of KW-13* in vivo*.

## Figures and Tables

**Table 1:** The sequences of six designed peptides.

Name	Sequence	Design
KWKK-13	KWKKPKLLKKLLK	N-terminal domain contains three cationic lysine residues and C-terminal is a standard *α*-helix (KLLK). They are connected with proline.

KW-13	KWKYPKLLKKLLK	N-terminal domain contains two cationic lysine residues and C-terminal is a standard *α*-helix (KLLK). They are connected with proline.

RFFR-15	RRWWRFPRFFRRFFR-NH2	N-terminal domain contains three cationic arginines and C-terminal is a standard *α*-helix (RFFR). They are connected with proline. C-terminal amidation stabilizes the peptide.

RFPP-18	RRWWRFPPPRFPPRFPPP-NH2	N-terminal domain contains three cationic arginines and C-terminal is a standard *α*-helix (RFPP). They are connected with proline. C-terminal amidation stabilizes the peptide. This sequence contains more proline.

KPV-13	KWKLFKKIWGKPV-NH2	Hybrid peptide based on the AMPs in the database. The N-terminal is from cecropin A1 and C-terminal is from MSH.

KPV-8	KFRWGKPV-NH2	Hybrid peptide based on the AMPs in the database. The N-terminal is from cecropin A1 and C-terminal is from MSH.

**Table 2:** Physicochemical properties of KW-13 peptide.

Primary structure	KWKYPKLLKKLLK
Hydrophobic amino acid	I: 0, V: 0, L: 0, F: 0, C: 0, A: 0, W: 1
MW (Da)	1686.02
Cationicity	6
Total hydrophobic ratio	38%
Protein-binding potential (kcal/mol)	0.87

**Table 3:** Bacterial and fungi strains used in the current study.

Strain	Culture condition (°C)	Medium
*Staphylococcus aureus*	37	Luria-Bertani
*Staphylococcus epidermidis *	37	Luria-Bertani
*Escherichia coli*	37	Luria-Bertani
*Klebsiella pneumoniae*	37	Luria-Bertani
*Pseudomonas aeruginosa*	37	Luria-Bertani
*Monilia albicans*	30	Martin Broth, Modified
*Aspergillus niger*	30	Martin Broth, Modified

Luria-Bertani (LB): peptone 10 g/L, yeast extract 5 g/L, and NaCl 10 g/L, pH 7.4 after sterilization; Martin Broth, Modified: peptone 10 g/L, yeast extract 2 g/L, glucose 20 g/L, dipotassium hydrogen phosphate 1 g/L, and MgSO_4_ 0.5 g/L, pH 6.4 ± 0.2 after sterilization.

**Table 4:** Peptides with homology to KW-13.

Peptide^a^	Source	Homology
KW-13	Synthetic, *de novo* design	
AP00143	Synthetic, *de novo* design	61.53%
AP00506	Synthesis	50%
AP00501	Synthesis	50%
AP00142	Synthetic, database-aided design using freq. occurring residues	50%
AP00859	Rana temporaria	46.15%

^a^The homologue peptides are identified according to APD ID.

**Table 5:** Antimicrobial activities (MIC) of peptide against bacteria and fungi.

Strain	MIC (*µ*g/mL)					
	KW-13	RFFR-15	RFPP-18	KWKK-13	KPV-13	KPV-8
*Staphylococcus aureus*	16	64	>512	>512	>512	>512
*Staphylococcus epidermidis*	4	128	512	>512	32	>512
*Escherichia coli*	64	256	128	256	512	>512
*Klebsiella aeruginosa*	128	>512	>512	>512	>512	>512
*Pseudomonas aeruginosa*	128	>512	256	>512	>512	>512
*Monilia albicans*	ND	ND	ND	ND	ND	ND
*Aspergillus niger*	256	>512	>512	>512	>512	>512

MIC: minimal inhibitory concentration. These concentrations represent mean values of three independent experiments performed in duplicate. ND denotes no inhibition was observed even at a peptide concentration of 512 *μ*g/mL.
